# Cold Atmospheric Pressure Plasma-Activated Medium Induces Selective Cell Death in Human Hepatocellular Carcinoma Cells Independently of Singlet Oxygen, Hydrogen Peroxide, Nitric Oxide and Nitrite/Nitrate

**DOI:** 10.3390/ijms22115548

**Published:** 2021-05-24

**Authors:** Yan Li, Tianyu Tang, Haejune Lee, Kiwon Song

**Affiliations:** 1 Department of Biochemistry, College of Life Science and Biotechnology, Yonsei University, Seoul 03722, Korea; liyan900506@hotmail.com; 2 Department of Electrical Engineering, Pusan National University, Busan 46241, Korea; tanghutu@hotmail.com (T.T.); haejune@pusan.ac.kr (H.L.)

**Keywords:** cold atmospheric pressure plasma (CAP), plasma-activated medium (PAM), hepatocellular carcinoma cell lines Hep3B and Huh7, reactive oxygen and nitrogen species (RONS), charged particles

## Abstract

Cold atmospheric pressure plasma (CAP) and plasma-activated medium (PAM) induce cell death in diverse cancer cells and may function as powerful anti-cancer agents. The main components responsible for the selective anti-cancer effects of CAP and PAM remain elusive. CAP or PAM induces selective cell death in hepatocellular carcinoma cell lines Hep3B and Huh7 containing populations with cancer stem cell markers. Here, we investigated the major component(s) of CAP and PAM for mediating the selective anti-proliferative effect on Hep3B and Huh7 cells. The anti-proliferative effect of CAP was mediated through the medium; however, the reactive oxygen species scavenger N-acetyl cysteine did not suppress PAM-induced cell death. Neither high concentrations of nitrite or nitrite/nitrate nor a low concentration of H_2_O_2_ present in the PAM containing sodium pyruvate affected the viability of Hep3B and Huh7 cells. Inhibitors of singlet oxygen, superoxide anions, and nitric oxide retained the capacity of PAM to induce anti-cancer effects. The anti-cancer effect was largely blocked in the PAM prepared by placing an aluminum metal mesh, but not a dielectric PVC mesh, between the plasma source and the medium. Hence, singlet oxygen, hydrogen peroxide, nitric oxide, and nitrite/nitrate are not the main factors responsible for PAM-mediated selective death in Hep3B and Huh7 cells. Other factors, such as charged particles including various ions in CAP and PAM, may induce selective anti-cancer effects in certain cancer cells.

## 1. Introduction

Cold atmospheric pressure plasma (CAP) and plasma-activated medium (PAM) are considered novel anti-cancer therapies based on studies showing that CAP and PAM selectively induce apoptosis in various cancer cells [1,2,3,4,5]. However, we still do not completely understand the molecular mechanism of the physiological effects of CAP and PAM, thereby delaying the development of CAP and PAM as clinical therapies. CAP is a partially ionized neutral gas containing complex and highly reactive chemical components [6,7]. Typically, CAP contains various ions, electrons, free radicals, and neutral molecules, including reactive oxygen species (ROS) and reactive nitrogen species (RNS) [1]. It primarily generates short-lived species such as hydroxyl radicals (·OH), nitric oxide radicals (·NO), superoxide radicals (O_2_^−^), atomic oxygen (O), singlet oxygen (^1^O_2_), and excited nitrogen (N*) [1,8]. CAP-generated short-lived species, such as ·OH and ·NO radicals, are converted to stable RONS, such as H_2_O_2_ and ·NO derivatives NO_2_^−^ and NO_3_^−^, in the PAM [8].

The ROS scavenger N-acetyl cysteine (NAC) inhibited CAP- and PAM-induced cell death in some cancer cells such as HeLa [5,9,10,11,12]. The study by Kurake et al. showed that H_2_O_2_ induced the death of cancer cells, and NO_2_^−^ enhanced the anti-cancer effect of H_2_O_2_ when NO_2_^−^ was added to the H_2_O_2_-containing medium [13]. The combined treatment of H_2_O_2_ and NO_2_^−^ exerts anti-cancer effects similar to that of PAM; however, the effect is not as strong as that of PAM [13]. Bauer et al. further demonstrated that H_2_O_2_ and NO_2_^−^ generated by CAP react to form ^1^O_2_ to inactivate some membrane-associated catalase molecules in tumor cells. This allows the formation of secondary ^1^O_2_ by the interaction between H_2_O_2_ and ONOO^−^ for optimal inactivation of catalase to induce cancer-cell-specific death [14]. The ROS-induced apoptosis pathway is mediated by O_2_^−^ generated by NADPH oxidase 1 (Nox1) in cells [14]. These reports suggest that reactive oxygen and nitrogen species (RONS) that are generated by CAP and are present in PAM, including H_2_O_2_, play critical roles in mediating the anti-proliferative effect of CAP and PAM.

In contrast, several other studies have shown that cell death induced by CAP and PAM is ROS-independent. The ROS scavenger NAC, which suppresses the CAP-induced cytotoxic effect in HeLa cells, did not suppress the anti-proliferative effect of CAP in U373MG glioma cells [9]. It demonstrated that U373MG cells have a higher antioxidant activity than HeLa cells, and an ROS-independent cell death is induced by CAP in U373MG cells [9]. Moreover, the ROS scavenger NAC or catalase did not suppress plasma-induced cell death in MDA-MB-231 breast cancer cells [10]. This showed that CAP can significantly inhibit IL-6R/pSTAT3 activation to reduce cell proliferation independently of ROS-related pathways [10]. Additionally, Tornin et al. showed that a high concentration of H_2_O_2_ generated in the PAM without pyruvate induces apoptosis, but the PAM containing pyruvate, a scavenger of H_2_O_2_, can also induce the death of osteosarcoma cells [15]. These reports suggest that other mechanisms independent of ROS may also play a role in the plasma-mediated anti-proliferative effect. Thus, the anti-proliferative effect of ROS in CAP and PAM in different cancer cells remains controversial.

Nitric oxide (·NO) has also been reported to induce cell death in cancer cells through various pathways [16,17]. CAP-activated ·NO water selectively induces apoptosis in HeLa cells by facilitating the accumulation of RONS in cells [18]. ·NO is converted to NO_2_^−^, which enhances the cytotoxic effect of H_2_O_2_ [13]. However, the anti-cancer effect of ·NO is controversial because ·NO plays a dual role in accelerating or inhibiting the proliferation of cancer cells [16]. The function of ·NO in cancer cells depends on the concentration and exposure duration of ·NO and the cellular sensitivity to ·NO [16,17]. These reports suggest that the functions of RONS in CAP and PAM to induce cell death depend on experimental parameters such as the concentration and cell type. Thus, the component(s) and mechanisms of the anti-cancer effects of CAP and PAM remain elusive.

Hepatocellular carcinoma (HCC) is a major histological subtype of primary liver cancer, with a high rate of recurrence and heterogeneity [19]. Known anti-cancer methods have not been successful in inducing cell death in HCC. In a previous study, we showed that CAP or PAM induced selective cell death in HCC cell lines Hep3B and Huh7 containing populations of cells with cancer stem cell markers [20]. In this study, we examined the major component(s) of CAP and PAM responsible for mediating the selective anti-proliferative effect on Hep3B and Huh7 cells to elucidate the mechanism underlying the cell death induced by CAP and PAM.

## 2. Results

### 2.1. The Anti-Proliferative Effect of CAP Is Mediated by PAM

**Figure 1:**
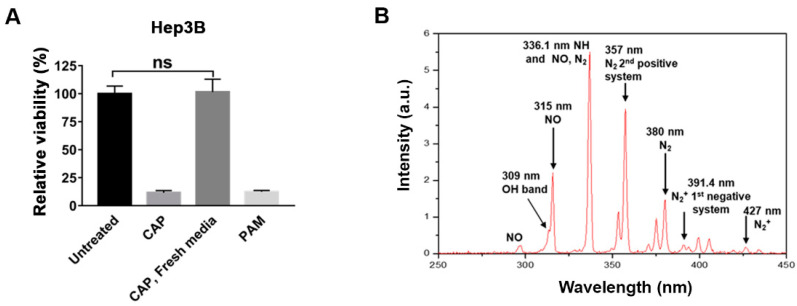
The anti-proliferative effect of CAP is dependent on the PAM. (**A**) Hep3B cells were seeded in 35 mm culture dishes and incubated for 18 h before various treatments. The cells were treated with CAP for 2.5 min or PAM, or exposed to CAP for 2.5 min followed by an immediate switch to fresh DMEM. Cell viability was measured by the MTT assay, and the relative viability was calculated as the ratio of the viability of treated cells to the viability of untreated cells at 72 h after the treatment. The results are presented as the mean ± SD of at least three independent experiments. ns, not significant. (**B**) The components generated by the CAP device were analyzed by OES.

In a previous study, we showed that CAP remarkably decreased the viability of Hep3B and Huh7 cells [20]. The anti-proliferative effect of PAM was as effective as that of CAP when Hep3B and Huh7 cells were treated with CAP for 2.5 min or the PAM prepared by exposing the fresh medium with CAP for 2.5 min (Figure 1A and Supplementary Figure S2A). We described the source of CAP and detailed conditions of the CAP, including the schematic of the device, in the previous study [20], and briefly address it here in the Materials and Methods. In this study, we investigate which components generated by CAP and transferred to the medium for PAM are responsible for the selective anti-proliferative effect of PAM. We first tested whether the anti-proliferative effect of CAP is mediated by the CAP-exposed medium PAM. We treated Hep3B and Huh7 cells with CAP for 2.5 min followed by an immediate switch to fresh medium after CAP exposure, and compared their viability with that of CAP- or PAM-exposed cells. The relative viability of these cells was similar to that of the untreated control cells at 72 h after treatment (Figure 1A and Supplementary Figure S2A). These results demonstrated that the anti-proliferative effect of CAP was mediated via the components generated by CAP and transferred to the medium, which are present in PAM.

We then evaluated the molecules generated from the CAP device using optical emission spectroscopy (OES). In this study, we used an s-DBD device to generate CAP through the breakdown of air. We observed dominant emission peaks from N-related molecular bands between 283 and 380 nm, which can likely be attributed to the nitrogen molecules occupying the dominant fraction of air (78%) and dissociated by the strong surface DBD plasma (Figure 1B). In addition to these lines, many other lines were observed for the N_2_^+^ emission band between 391 and 428 nm and the OH band at 309 nm (Figure 1B). At the DBD surface, water molecules are also dissociated with OH and H because of the high electron temperature attributed to the discharge. The hydrogen atoms combine with the nitrogen atoms to form NH at a location far from the surface of the DBD as the electron temperature decreases. These short-lived species, such as ·OH and ·NO, generated in the discharge region are likely to convert to long-lived species such as H_2_O_2_, NO_2_^−^, and NO_3_^−^ in PAM. Thus, the OES results suggested that N_2_^+^, ·OH, ·NO, and RONS, such as H_2_O_2_, NO_2_^−^, and NO_3_^−^, are the main components of PAM.

### 2.2. The ROS Scavenger NAC Did Not Suppress PAM-Induced Cell Death in Hep3B Cells

**Figure 2:**
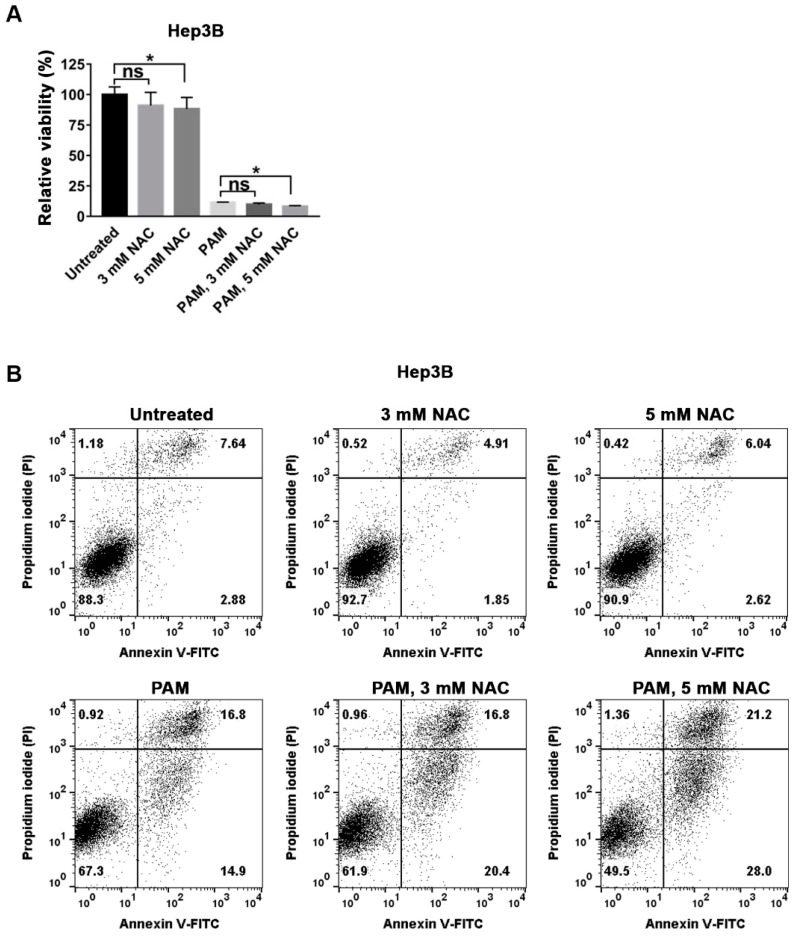
The ROS scavenger NAC did not suppress PAM-induced cell death in Hep3B cells (**A**,**B**) The effect of NAC, an ROS scavenger, on the anti-proliferative activity of PAM was examined in Hep3B cells. Hep3B cells were treated with 3 mM NAC, 5 mM NAC, PAM, and PAM containing 3 mM or 5 mM NAC. (**A**) Cell viability was measured by the MTT assay, and the relative viability was calculated as the ratio of the viability of treated cells to the viability of untreated cells at 72 h after the treatment. The results are presented as the mean ± SD of at least three independent experiments. * *p* < 0.05 indicates significant difference; ns, not significant. (**B**) Death of the Hep3B cells shown in (**A**) was examined using flow cytometry after staining the cells with Annexin V-FITC and PI.

Previous studies have shown that RONS generated in PAM are the main mediators of the anti-proliferative effect of PAM [13,14]. To confirm the role of ROS in mediating the anti-proliferative effect of PAM on Hep3B and Huh7 cells, we treated the cells with PAM in the presence of N-acetyl-L-cysteine (NAC), a typical ROS scavenger. We first examined whether the treatment of Hep3B cells with only 3 or 5 mM NAC affected cell viability. Three mM NAC did not affect the viability of Hep3B cells, as shown by both the MTT assay (Figure 2A) and flow cytometric analysis (Figure 2B). Treatment of Hep3B cells with 5 mM NAC slightly decreased the viability compared to that of the untreated control, as shown by MTT assay, and no cell death was detected by flow cytometry (Figure 2A,B). PAM was prepared by CAP exposure and then mixed with 3 or 5 mM NAC to treat the cells. The presence of 3 or 5 mM of NAC in PAM did not suppress the decreased viability mediated by PAM in Hep3B cells, as shown by both MTT assay and flow cytometry (Figure 2A,B). The subset of dead cells even increased when Hep3B cells were co-treated with PAM and 5 mM NAC, compared with the cells treated with PAM alone (Figure 2B). Similar to Hep3B, the presence of 3 mM or 5 mM NAC in PAM did not suppress the anti-proliferative effect of PAM in Huh7 cells (Supplementary Figure S2B). These results suggest that ROS may not mediate the PAM-induced anti-proliferative effect in HCC cell lines Hep3B and Huh7.

### 2.3. The Concentration of H_2_O_2_ Present in the PAM With Pyruvate Did Not Induce Cell Death in Hep3B Cells

**Figure 3:**
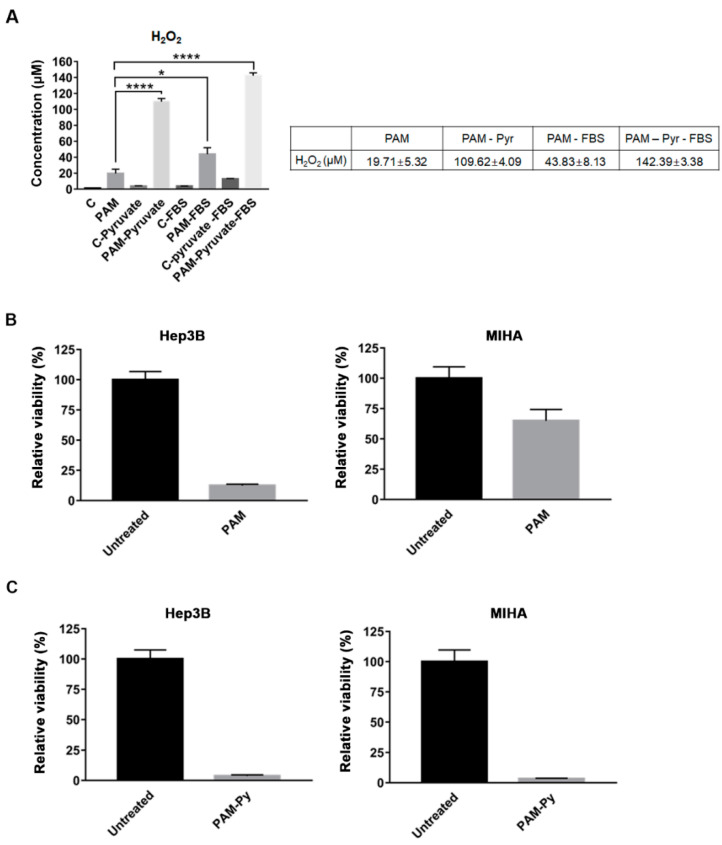
H_2_O_2_ concentrations in the PAM with and without pyruvate and their effects on Hep3B cells. (**A**) Concentrations of H_2_O_2_ were measured immediately after CAP exposure for 2.5 min in the PAM with both of 1mM sodium pyruvate (Pyr) and 10% FBS, only with 1mM Pyr or 10% FBS, and without both 1 mM Pyr and 10% FBS H_2_O_2_ using Amplex^®^ Red Hydrogen Peroxide assay. (**B**,**C**) Hep3B cells seeded and pre-incubated for 18 h were treated with (**B**) PAM and (**C**) PAM without 1mM pyruvate (PAM-Py). Cell viability was measured using the MTT assay, and the relative viability was calculated as the ratio of the viability of treated cells to the viability of untreated cells at 72 h after the treatment. (**A**–**C**) The results are presented as the mean ± SD of at least three independent experiments. * *p* < 0.05, and **** *p* < 0.0001 indicate significant difference; ns, not significant.

H_2_O_2_ has been reported to act as the main anti-cancer factor in PAM when using a culture medium devoid of sodium pyruvate (Pyr), a scavenger of H_2_O_2_ [13]. To examine the role of H_2_O_2_ in the anti-proliferative effect of PAM on Hep3B and Huh7 cells, we directly measured the concentrations of H_2_O_2_ in PAM prepared with different culture medium formulations. We found that 19.71 μM of H_2_O_2_ was present in the PAM used in this study, where both 1 mM Pyr and 10% FBS were added to the medium (Figure 3A). H_2_O_2_ with a concentration of 43.83 μM was present in the PAM, which was prepared in the absence of 10% FBS (Figure 3A). On the contrary, 109.62 μM and 142.39 μM of H_2_O_2_ were respectively detected in the PAM prepared in the absence of Pyr or both Pyr and FBS (Figure 3A). These results showed that 1 mM Pyr in the medium scavenged high concentrations of H_2_O_2_ generated in PAM, as previously reported [21]. Consistent with the study conducted by Boehm et al. showing that FBS also scavenges H_2_O_2_ [22], our observations showed that the concentration of H_2_O_2_ was higher in the PAM without FBS than in the PAM containing FBS, demonstrating that FBS also acts as a scavenger of H_2_O_2_ in PAM. Thus, the PAM used to induce death of Hep3B and Huh7 cells contained relatively low concentrations of H_2_O_2_ owing to the scavenging effects of pyruvate and FBS in the medium.

To verify that H_2_O_2_ is not the main factor responsible for the death of Hep3B cells mediated by the PAM used in this study, we directly treated Hep3B cells with 142 μM H_2_O_2_ (the same concentration of H_2_O_2_ generated by CAP in the absence of pyruvate and FBS) in a medium containing pyruvate and FBS, and compared the viability with that of PAM-treated cells. Consistently, we observed that H_2_O_2_ did not affect the cell viability with the presence of pyruvate and FBS in the medium (Figure 5B), suggesting that H_2_O_2_ generated in PAM was not the main cause of the anti-proliferative effect of the PAM observed on the Hep3B cells.

In our previous study, we observed that PAM selectively induced cell death in Hep3B and Huh7 cells, but not in the normal liver cell line MIHA [20], as shown in Figure 3B (Data from the supplementary materials of our previous report [20]). To examine whether H_2_O_2_ in PAM controls the selective anti-proliferative effect of PAM, we compared the viability of Hep3B cells after treatment with PAM prepared in the presence or absence of 1 mM Pyr. The PAM prepared without pyruvate showed a strong anti-proliferative effect not only on Hep3B cells, but also on MIHA cells (Figure 3C), while treatment with PAM including pyruvate decreased the viability remarkably in Hep3B cells and only slightly in MIHA cells. These results indicated that high concentrations of H_2_O_2_ in the PAM lacking pyruvate exerted a strong anti-proliferative effect on both Hep3B and MIHA cells, demonstrating that cell death depends on H_2_O_2_ in the PAM without pyruvate. However, PAM without pyruvate did not demonstrate selectivity in exerting the anti-proliferative effect between Hep3B and MIHA cell lines, which was observed in PAM with pyruvate. Altogether, these results suggest that other factors, but not H_2_O_2_, control the selective anti-cancer effect of PAM with pyruvate.

### 2.4. The Inhibitors of ^1^O_2_ and O_2_^−^ Did Not Suppress the Anti-Proliferative Effect of PAM

**Figure 4:**
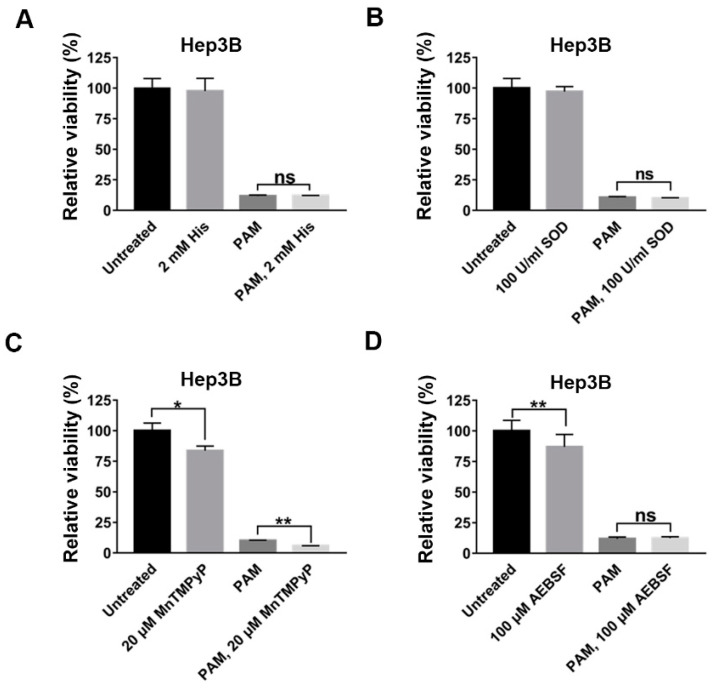
Inhibitors of ^1^O_2_ or O_2_^−^did not suppress the PAM-induced anti-cancer effect on Hep3B cells. (**A**–**D**) Hep3B cells were seeded in 35 mm culture dishes and incubated for 18 h. Prior to PAM treatment, the cells were pre-incubated with the inhibitors for 1 h. Hep3B cells were treated by PAM with the (**A**) ^1^O_2_ scavenger, 2 mM histidine, (**B**) 100 U/mL SOD, (**C**) SOD mimetic, 20 μM MnTMPyP, (**D**) NOX1 inhibitor, 100 μM AEBSF. (**A**–**D**) Cell viability was analyzed at 72 h after the PAM treatment using the MTT assay. The relative viability was calculated as the ratio of the viability of treated cells to that of untreated cells at 72 h. Untreated cells incubated for the same intervals were used as negative controls. The results are plotted as mean ± SD of three independent experiments. * *p* < 0.05 and ** *p* < 0.01 indicate significant difference; ns, not significant.

Bauer et al. showed that ^1^O_2_ and O_2_**^−^** contributed to the induction of cancer cell death [14]. H_2_O_2_ and NO_2_^−^ generated in PAM were induced to form primary ^1^O_2_ and activated ROS-dependent cell death pathways. O_2_**^−^** generated by Nox1 in cells was also involved in the ROS cell death pathways [14]. They showed that the inhibitors of ^1^O_2_ or O_2_**^−^** completely suppressed the cell death effect of PAM [14]. Considering that NADPH oxidase 1 (Nox1), which mediates ROS generation, is also present in Hep3B and Huh7 cells [23], we tried to examine whether ^1^O_2_ and O_2_**^−^** also contribute the selective cell death of Hep3B and Huh7 by PAM. Moreover, Tornin et al. showed that PAM containing pyruvate induced cell death in osteosarcoma and suggested that other ROS may be crucial for PAM-mediated cell death [15]. Thus, we wanted to investigate whether other ROS, which cannot be scavenged by NAC or pyruvate, exert a cytotoxic effect on Hep3B and Huh7 cells. We first examined the anti-proliferative function of ^1^O_2_ using histidine as a scavenger. We evaluated whether treatment with 2 mM histidine inhibited the anti-cancer effect of PAM on Hep3B cells. The presence of histidine itself did not affect the viability of Hep3B cells compared to that observed in the untreated control (Figure 4A). When Hep3B cells were treated with PAM and 2mM histidine together, the presence of histidine did not affect the PAM-induced anti-proliferative effect on Hep3B cells (Figure 4A). Next, we examined whether O_2_^−^ is an important factor responsible for the anti-proliferative effect of PAM with superoxide dismutase (SOD) or SOD mimetics. When 100 U/mL SOD or an SOD mimetic, 20 μM MnTMPyP, was added to the PAM, they did not block the PAM-induced anti-cancer effect on Hep3B cells (Figure 4B,C). In addition, considering the report that PAM-derived species react with membrane-associated NADPH oxidase, NOX1, resulting in high concentrations of extracellular O_2_**^−^** [14], we also treated Hep3B cells with PAM added to the NOX1 inhibitor AEBSF (100 μM). The presence of 100 μM AEBSF in PAM did not suppress the anti-cancer effect of PAM on Hep3B cells (Figure 4D).

We treated another HCC cell line, Huh7, with these inhibitors in conjunction with PAM. These ^1^O_2_ or O_2_**^−^** inhibitors did not suppress the PAM-induced anti-proliferative effect on Huh7 cells (Supplementary Figure S2C–E). Since the treatment of Hep3B and Huh7 cells with the suggested concentrations of AEBSF (100 μM) and MnTMPyP (20 μM) slightly affected the viability of cells compared to that observed in the untreated cells (Figure 4C,D, Supplementary Figure S2D,E), we also examined their effects at reduced concentrations. We observed consistent results showing that they did not inhibit the PAM-induced anti-proliferative effect on Hep3B and Huh7 cells (Supplementary Figures S1 and S2D,E).

Taken together, we observed that PAM retained its anti-proliferative effect on Hep3B and Huh7 cells in the presence of an inhibitor of singlet oxygen or superoxide anions. These results strongly suggest that neither singlet oxygen nor superoxide anions are the main contributors to the anti-proliferative effect of PAM in Hep3B and Huh7 cells, and other factors are likely involved in PAM-induced cell death.

### 2.5. NO Scavenger Did Not Suppress the Anti-Proliferative Effect of PAM on Hep3B Cells

**Figure 5:**
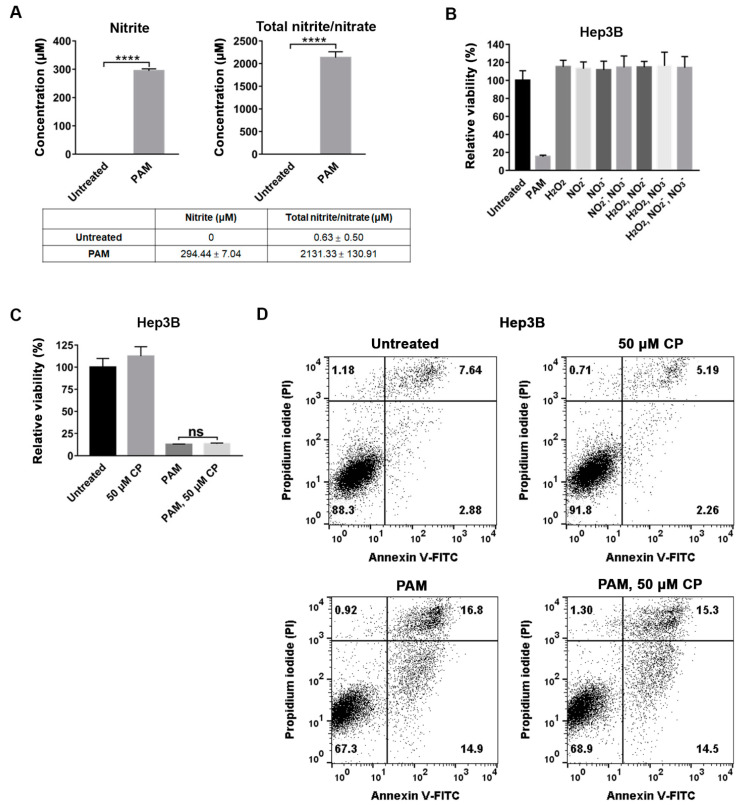
RNS are not the main factors responsible for the PAM-induced death in Hep3B cells. (**A**) The concentration of NO_2_^−^ and NO_3_^−^ in the PAM was measured immediately after CAP exposure for 2.5 min. The concentrations of NO_2_^−^ and NO_3_^−^ were detected using the Griess assay. (**B**) The effect of NO_2_^−^, NO_3_^−^, and H_2_O_2_ individually or in combination on the viability of Hep3B cells was compared to those observed on the PAM-treated and the untreated control. Concentrations of 142 μM of H_2_O_2_, 294 μM of nitrite, and 1.8 mM of nitrate were used. (**C**,**D**) The effect of carboxy-PTIO (CP), an NO scavenger, on the anti-proliferative activity of PAM in Hep3B cells was analyzed. Hep3B cells were treated with PAM, 50 μM CP, or PAM immediately followed by 50 μM CP. Untreated cells served as the negative control. (**B**,**C**) The cells were incubated for 72 h after the initial treatment, and cell viability was measured using the MTT assay. Relative viability was calculated as the ratio of the viability of treated cells to that of the untreated cells at 72 h. Each experiment was repeated thrice, and the data are presented as mean ±  SD. **** *p* < 0.0001 indicate significant differences; ns, not significant. (**D**) Cell death was monitored as shown in (**C**) by flow cytometry after staining with Annexin V-FITC and PI.

Having observed that the anti-proliferative effect of PAM on Hep3B cells was not mediated by H_2_O_2,_ we analyzed the effect of NO_2_^−^ and NO_3_^−^ as the concentrations of NO_2_^−^ and NO_3_^−^ were significantly increased in the PAM. Compared to the CAP-unexposed medium, approximately 294 μM of NO_2_^−^ and 2.1 mM of total NO_2_^−^/NO_3_^−^ were detected in the PAM used in this study (Figure 5A). The combined treatment of H_2_O_2_ and NO_2_^−^ demonstrates an anti-cancer effect similar to that of PAM [13]. To verify whether NO_2_^−^ and NO_3_^−^, and their combination with H_2_O_2_, were the main factors responsible for the anti-proliferative effect of the PAM on Hep3B cells, 294 μM of NO_2_^−^, 1.8 mM of NO_3_^−^ (the concentration of NO_2_^−^ and NO_3_^−^ measured in the PAM, Figure 5A), and 142 μM of H_2_O_2_ (the H_2_O_2_ concentration measured in the PAM without pyruvate and FBS) were directly dissolved in the culture medium containing pyruvate and FBS individually or combined, and the viability of their treatments was compared with that of the PAM-treated Hep3B cells. Unexpectedly, the single or combined treatment of NO_2_^−^ and NO_3_^−^, as well as their combination with H_2_O_2_, did not affect the viability of Hep3B cells, while the PAM-treated Hep3B cells showed a remarkable decrease in viability (Figure 5B).

Since our OES analysis of the CAP showed that a peak corresponding to ·NO (Figure 1B), and high concentrations of NO_2_^−^ and NO_3_^−^, which mediate the synthesis of ·NO, were present in the PAM, we expected that a high concentration of ·NO might be present in the PAM used to treat Hep3B cells. ·NO is relatively stable in aqueous solutions with a half-life of up to 24 h [24]. In addition, previous reports suggest that ·NO causes selective apoptosis in tumor cells through various pathways [16,18]. Thus, we examined whether ·NO is directly responsible for the anti-proliferative effect of PAM. To examine the anti-proliferative effect of ·NO in PAM, we added carboxy-PTIO (CP), an ·NO scavenger, to the PAM to treat Hep3B cells, and observed whether the presence of CP in PAM suppressed the anti-proliferative effect. A 50 μM concentration of CP was used, as this concentration of CP did not affect cell proliferation (Figure 5C). When we compared the viability of Hep3B cells treated with CP alone, PAM, or PAM followed by CP, the presence of CP did not reduce PAM-induced cell death in Hep3B cells (Figure 5C,D). Taken together, these results strongly suggest that NO_2_^−^, NO_3_^−^, or·NO are not the major factors responsible for PAM-induced death in Hep3B cells.

### 2.6. Aluminum Mesh, Not PVC Mesh, Remarkably Decreased the Anti-Cancer Effect of PAM

Previously, Lee et al. reported that the physiological effect of plasma was blocked when using a grounded metal mesh, and suggested that the negative charges of radicals might have an important role in plasma-mediated effects [25]. The presence of a grounded metal mesh influences the electric potential profile to prevent only the negative charges from passing through the mesh [25]. Because the mesh location is far from the s-DBD, which generates plasma only on the surface, the ground electrode does not change the amount of radical generation. Thus, we verified whether the negatively charged particles, including ions from CAP, play a critical role in the anti-proliferative effect of PAM on Hep3B cells. To examine the effect of negatively charged molecules in PAM, an aluminum (Al) mesh was used. A dielectric PVC mesh was also tested to eliminate the impact of the reduced open area. A dielectric PVC mesh or Al mesh was placed between the CAP and the medium when the PAM was prepared. The Al mesh forms a potential well to entrap negative charges, and the PAM is not provided with negatively charged molecules from the CAP. In contrast, the dielectric PVC mesh cannot hinder the negatively charged molecules from passing through the mesh.

**Figure 6:**
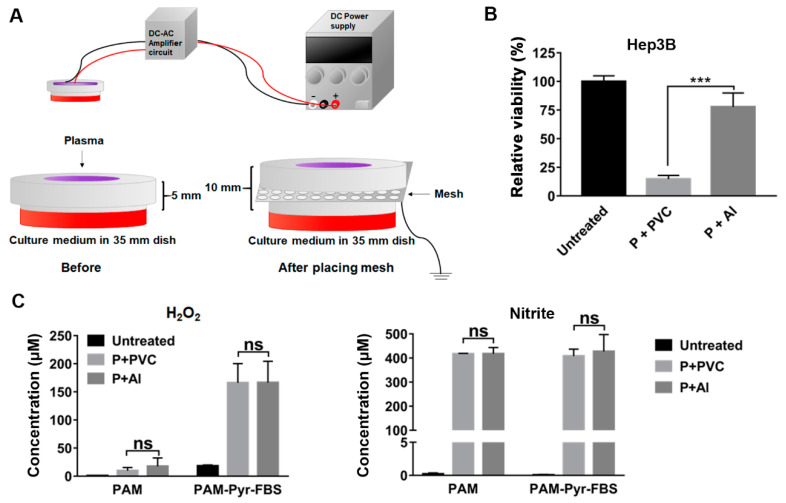
Aluminum mesh, but not PVC mesh, significantly diminished the anti-proliferative effect of PAM. (**A**,**B**) The PAM was prepared by placing aluminum mesh (d, 1.5mm; t, 0.5 mm), PVC mesh (d, 1.2mm; t, 0.5 mm), or no mesh, respectively, and was treated with Hep3B cells; d, hole diameter; t, mesh thickness. (**A**) Schematic diagram of the preparation of PAM in the presence of mesh. (**B**) The viability of cells following each PAM treatment was measured after 72 h of the treatment using MTT assays. Relative viability was calculated as the ratio of the viability of treated to untreated cells observed at 72 h. (**C**) Concentrations of H_2_O_2_ and NO_2_^−^ were measured in the PAM immediately after CAP exposure with PVC or Al metal mesh. The PAM containing both 1 mM sodium pyruvate (Pyr) and 10% FBS or without both 1 mM Pyr and 10% FBS was used. The concentrations of H_2_O_2_ were detected using Amplex^®^ Red Hydrogen Peroxide assay and the concentrations of NO_2_^−^ were detected using the Griess assay. Untreated culture medium was used as a control. (**B**,**C**) The results are plotted as mean ±  SD of at least three independent experiments. *** *p* < 0.001 indicate significant difference; ns, not significant.

When we generated PAM in this study, the distance between the plasma source and the medium surface was fixed at 5 mm. However, to place the mesh between the plasma source installed in each dish cover and the surface of the culture medium, the distance between the plasma source and the medium surface was maintained at 10 mm (Figure 6A). As the distance from the plasma source to the surface of the culture medium increased to 10 mm, the exposure time of plasma to make the PAM was extended to produce PAM showing the same anti-proliferative effect on Hep3B cells as that observed with the PAM employed in previous experiments in this study. We first verified that the PAM prepared by exposure to CAP at a 10 mm distance decreased the viability of Hep3B as the PAM used previously while maintaining its selective anti-proliferative activity on Hep3B and Huh7 cells but not on MIHA cells. Following 6.5 min of CAP exposure at 10 mm, PAM showed decreased viability and selectivity on Hep3B and Huh7 cells (data not shown). Then, we treated Hep3B cells with PAM prepared in the presence of Al or PVC mesh and compared the anti-proliferative effects with those observed with the PAM produced without the mesh. We found that the PAM-induced anti-proliferative effect was reduced significantly while using PAM produced with the Al-grounded metal mesh. However, no significant difference was observed when using PAM produced with the PVC mesh (Figure 6B). The viability of Hep3B cells recovered to approximately 75% of the untreated cells when the PAM was prepared with an Al-grounded mesh (Figure 6B). When we examined the anti-proliferative effect of PAM using Al or PVC mesh on Huh7 cells, we also found that only PAM produced using Al lost its anti-proliferative effect (Supplementary Figure S2F). These results strongly suggest that negatively charged particles, including various ions, play a major role in mediating the anti-cancer effects of PAM.

In order to verify that the different anti-proliferative effects of PAM produced using Al and PVC cannot be attributed to the different concentrations of H_2_O_2_ and/or nitrite, we measured their concentrations in the PAM produced with Al and PVC mesh, respectively. Consistent with our results shown in Figure 3 and Figure 5, the PAM prepared with Al and PVC, demonstrating a large discrepancy in its anti-proliferative effect on Hep3B and Huh7 cells, did not show much difference in the concentrations of H_2_O_2_ and nitrite (Figure 6C). These observations further confirm that H_2_O_2_ and/or nitrite are not the critical factors responsible for the selective anti-proliferative effect of PAM.

## 3. Discussion

The anti-cancer effects of CAP and PAM have been demonstrated in many studies performed with different cancer cells. However, for the safe clinical application of CAP and PAM in anti-cancer therapies, the active components of CAP and PAM and the underlying mechanism responsible for the selective anti-cancer effect should be elucidated. Hepatocellular carcinoma (HCC) is a major histological subtype of primary liver cancer and the third leading cause of cancer-related deaths worldwide [19]. Known anti-cancer treatment methods have not been successful in inducing cell death in HCC. However, we showed CAP- and PAM-mediated selective cell death in Hep3B and Huh7 cell lines of HCC containing populations of cells with cancer stem cell markers, but not in the normal hepatocyte cell line MIHA [20]. In this study, we attempted to elucidate the component(s) responsible for the anti-proliferative effect of CAP and PAM to support the development of PAM as a main or auxiliary therapy for HCC in the future.

Many studies have reported that RONS present in CAP and PAM play key roles in inducing death in various cancer cells. For example, the ROS scavenger NAC inhibited CAP- and PAM-induced cell death in U87MG glioblastoma cells, HCT-116 colorectal carcinoma cells [11], HeLa cells [5,9], and MDA-MB-453 breast cancer cells [10]. Kurake et al. demonstrated that treatment with H_2_O_2_ induced the death of U251SP human glioblastoma cells, and NO_2_^−^ enhanced the anti-cancer effect of H_2_O_2_ when added to the H_2_O_2_-containing medium, suggesting that H_2_O_2_ plays a major role in CAP- and PAM-induced anti-cancer effects [13]. However, several other studies have shown that ROS-independent cell death is induced by plasma. The ROS scavenger NAC or catalase did not suppress plasma-induced cell death in U373MG glioma [9] or MDA-MB-231 breast cancer cells [10]. Conway et al. reported that ROS-independent cell death is induced by CAP in U373MG cells due to its antioxidant activity in U373MG cells [9]. Moreover, Liu et al. showed that CAP can inhibit IL-6R/pSTAT3 activation to reduce cell proliferation independently of ROS-related pathways in MDA-MB-231 breast cancer cells [10]. Additionally, Tornin et al. demonstrated that PAM containing pyruvate remarkably reduced H_2_O_2_ concentration and ROS generation, but still induced cell death in osteosarcoma [15].

Thus, we tested the effects of most of the reported components of CAP and PAM to deduce the component(s) responsible for the selective anti-cancer effect of PAM in Hep3B and Huh7 cells. We used DMEM culture medium containing pyruvate in this study, which is a typical medium used for mammalian culture systems. We found that the anti-proliferative function of CAP was mediated via the medium, suggesting that the component(s) of PAM directly transmitted from the CAP is/are responsible for the anti-cancer effect. We found that treatment with NAC did not inhibit PAM-induced cell death in Hep3B and Huh7 cells. Consistently, a low concentration of H_2_O_2_ was detected in the PAM containing pyruvate, and it did not decrease the viability of Hep3B or Huh7 cells. Treatment with the inhibitors of singlet oxygen or superoxide anions did not suppress the anti-proliferative effect of PAM on Hep3B or Huh7 cells. Although high concentrations of NO_2_^−^ and NO_3_^−^ were detected in the PAM (Figure 5A), we also observed that the single or combined treatment with H_2_O_2_, NO_2_^−^, and NO_3_^−^ did not affect the viability of Hep3B cells (Figure 5B). Moreover, even in the presence of CP, a ·NO scavenger, PAM induced death in Hep3B cells (Figure 5), although a strong peak attributed to ·NO was detected in the OES analysis of CAP. These results suggest that well-known RONS such as H_2_O_2_, singlet oxygen, superoxide anions, nitric oxide, and nitrate/nitrite are not the main factors responsible for the selective anti-proliferative effect of PAM on Hep3B and Huh7 cells.

In our results, PAM showed a strong anti-cancer effect in the absence of pyruvate in the medium, supporting previous reports demonstrating that H_2_O_2_ is responsible for CAP- or PAM-induced cell death in cancer. As suggested by Bauer et al., the subsequent reactions of H_2_O_2_ to generate singlet oxygen and superoxide anions might occur in this condition. However, we showed that PAM prepared without pyruvate did not show selective anti-proliferative activity on HCC cell line Hep3B and normal hepatocyte cell line MIHA, while PAM with pyruvate showed a selective effect on Hep3B cells. In addition, we observed that NAC partly recovered the reduced viability of MIHA cells treated with PAM (Supplementary Figure S3A). Furthermore, the same concentration of H_2_O_2_ generated by CAP added to the medium containing pyruvate partly decreased the viability of MIHA cells (Supplementary Figure S3B). These results support our conclusion that H_2_O_2_ of PAM may induce cell death regardless of cell type at high concentrations, but it is not responsible for the selective anti-proliferative effect of PAM observed on Hep3B and Huh7 cells.

To our surprise, PAM-induced anti-cancer effects observed on Hep3B and Huh7 cells were largely reduced when an Al-grounded metal mesh was placed between the plasma source and the medium during the preparation of PAM; however, placing the PVC mesh did not affect the anti-proliferative effect of PAM on these cells (Figure 6). Although they examined the different physiological effects of CAP, Lee et al. also reported that the plasma effect was inhibited by a grounded metal mesh [25]. These observations suggest that charged particles and ions, but not neutral radicals, might be crucial for plasma-mediated physiological outcomes observed in various cells, including the selective anti-cancer effect of CAP and PAM on Hep3B and Huh7 cells. The charged particles and ions generated by plasma but trapped by the metal-grounded mesh should be identified to understand the physiological effects of CAP and PAM in future studies.

Recent studies, including ours, strongly suggest that various biological effects of CAP and PAM are the outcome of complicated interactions between the component(s) of CAP and PAM and the physiology of different cell types. It would be difficult to deduce a single mechanism responsible for the selective anti-cancer effect observed in many different cancer cells. Thus, further studies on the molecular mechanisms, specificity, and conditions for CAP and PAM treatment in different types of cancer cells are necessary to develop CAP and PAM for clinical applications.

## 4. Materials and Methods

### 4.1. The CAP-Generating Device Used and the Optical Emission Spectroscopy (OES) of the Plasma Generated with Air from the Device

An s-DBD device, described in our previous report [20], was used to break down air with a relatively low voltage by utilizing electrode patterns on a dielectric surface covering the other electrode. A voltage of 4.7 kV and discharge power of 0.87 W were used to generate plasma. The thermal effect by the DBD is negligible because the temperature of the surface DBD is less than 36 degrees Celsius at a driving voltage of 4.7 kV. This CAP-generating device was designed to fit a 35 mm culture dish, as shown in Figure 6, so that the various chemical species generated by the CAP evenly covered the surface of the culture medium. The light spectrum of the plasma from this device was measured using OES in the wavelength range of 250–450 nm using a spectrometer (USB2000+, Ocean Optics, Largo, Dunedin, FL, USA) with an entrance slit of 25 μm. The exposure time was 0.8 μs, and the distance from the panel surface to the optical fiber was 15 mm.

### 4.2. Cell Culture

The HCC cell lines Hep3B (from the Korean Cell Line Bank, Seoul, Korea) and Huh7 (provided by Professor Young Nyun Park, College of Medicine, Yonsei University, Seoul, Korea), and the immortalized normal hepatocyte cell line MIHA (provided by Professor Suk Woo Nam, Catholic University, Seoul, Korea) were maintained in high glucose Dulbecco’s modified Eagle’s medium (DMEM; Gibco, Brooklyn, NY, USA) including 1 mM sodium pyruvate, 10% (*v*/*v*) fetal bovine serum (FBS; Gibco), and 1% antibiotic-antimycotic (Gibco) at 37 °C in an atmosphere containing 5% CO_2_.

### 4.3. Cell Exposure to CAP and PAM

Hep3B, Huh7, and MIHA cells were seeded at a density of 10^5^ cells onto 35 mm dishes, incubated for 18 h, and then exposed to CAP for 2.5 min. For PAM treatment, 1.5 mL of DMEM (containing pyruvate) in each 35 mm dish was exposed to CAP for 2.5 min and added to the cells. Following CAP exposure or PAM treatment, the cells were incubated further for 72 h before testing cell viability.

### 4.4. Cell Vviability Assay

Cell viability was analyzed using the 3-(4,5-dimethylthiazol-2-yl)-2,5-diphenyltetrazolium bromide (MTT; Sigma-Aldrich, St. Louis, MO, USA) assay. Cells treated with 1 mL of 0.5 mg/mL MTT solution were incubated for 1.5 h, and the resulting formazan was dissolved in 1 mL dimethyl sulfoxide (DMSO). Absorbance was measured at 570 nm using a microplate reader (SoftMax Pro 4.0, Molecular Devices, San Jose, CA, USA).

### 4.5. Flow Cytometry Analysis

To detect cell death, Hep3B cells were harvested 72 h after the initial treatment and stained with Annexin V-fluorescein isothiocyanate (Annexin V-FITC) and propidium iodide (PI). The cells were washed with PBS, resuspended in 1X binding buffer, and incubated with Annexin V-FITC and PI (BD Biosciences, San Jose, CA, USA) for 15 min according to the manufacturer’s protocol. After staining, 10,000 cells were analyzed per assay using a FACSCalibur (BD Biosciences) flow cytometer and FlowJo V10 software (BD Biosciences).

### 4.6. RONS Inhibitors

The final concentrations of 3 or 5 mM N-acetyl-L-cysteine (NAC; Sigma-Aldrich, St. Louis, MO, USA) dissolved in phosphate-buffered saline (PBS; pH 7.2), 50 μM carboxy-PTIO (CP; Sigma-Aldrich) in dimethyl sulfoxide (DMSO), histidine (Sigma-Aldrich) in distilled water, 4-(2-Aminoethyl) benzenesulfonyl fluoride (AEBSF; Sigma-Aldrich) in distilled water (pH adjusted to 7.2 before use), superoxide dismutase (SOD; Sigma-Aldrich) in 0.1 M potassium phosphate (pH 7.5), Mn(III)tetrakis(1-methyl-4-pyridyl)porphyrin, tetratosylate, and hydroxide (MnTMPyP; Sigma-Aldrich) in distilled water were adjusted in the medium for treatment.

### 4.7. Detection of the Concentration of H_2_O_2_ or Nitrite/Nitrate in PAM

The concentration of hydrogen peroxide in PAM was detected with Amplex Red reagent (10-acetyl-3,7-dihydroxyphenoxazine) using an Amplex^®^ Red Hydrogen Peroxide/Peroxidase Assay Kit (Invitrogen, Carlsbad, CA, USA). The concentration of nitrite/nitrate was detected by the Griess assay using the OxiSelect^TM^ nitric oxide (Nitrite/Nitrate) assay kit (Cell Biolabs, Inc., San Diego, CA, USA) according to the manufacturer’s protocol.

### 4.8. Effect of PAM Using Meshes

A dielectric PVC mesh and aluminum (Al) metal mesh were used in this experiment. To examine the effect of PAM on the mesh, each mesh was placed between the 35 mm dish and the plasma source installed in the cover; the distance from the source to the surface of the culture medium was 10 mm. The open-area ratio of the mesh was approximately 43%. A total of 1.5 mL of DMEM (containing pyruvate) was exposed to CAP for 6 min 30 sec with each mesh and used to treat the cells. Following PAM treatment, the cells were further incubated for 72 h, and cell viability was measured using the MTT assay.

### 4.9. Statistical Analyses

All data are expressed as the mean ± standard deviation (SD) of at least three independent experiments. Statistical analysis was performed for each experiment with Student’s *t*-test using GraphPad Prism (GraphPad Software Inc., San Diego, CA, USA). * *p* < 0.05 was considered statistically significant.

## 5. Conclusions

In this study, we demonstrated that plasma-induced selective cell death of HCC cell lines Hep3B and Huh7 cells depends on the medium, PAM. Treatment with the ROS scavenger NAC did not suppress PAM-induced cell death. Consistently, neither high concentrations of nitrite and nitrite/nitrate nor a low concentration of H_2_O_2_ present in PAM containing sodium pyruvate mediated the anti-proliferative effect of PAM. In addition, PAMs treated with the inhibitors of singlet oxygen, superoxide anions, and nitric oxide retained the capacity to induce anti-cancer effects in Hep3B and Huh7 cells. However, the anti-cancer effect of PAM is largely blocked by an Al-grounded metal mesh, but not by a dielectric PVC mesh. These findings suggest that known RONS, such as singlet oxygen, hydrogen peroxide, nitric oxide, and nitrite/nitrate are not the main causes of PAM-mediated selective cell death in Hep3B and Huh7 cells. The results indicate the involvement of other factors, such as negatively charged particles, in mediating the physiological effects in CAP and PAM.

## Supplementary Materials

The following are available online at https://www.mdpi.com/article/10.3390/ijms22115548/s1, Figure S1: The inhibitors against superoxide anions did not suppress PAM-induced anti-proliferation, Figure S2: Studies to identify the factors that affect viability in PAM-treated Huh7 cells, Figure S3: H_2_O_2_ in PAM decreased the viability of MIHA cells.
