# Supplementary material for: Cold Atmospheric Pressure Plasma-Activated Medium Induces Selective Cell Death in Human Hepatocellular Carcinoma Cells Independently of Singlet Oxygen, Hydrogen Peroxide, Nitric Oxide and Nitrite/Nitrate
Source: Int J Mol Sci. 2021 May 24;22(11):5548. doi: 10.3390/ijms22115548 (PMC8197405; doi:10.3390/ijms22115548)
Supplement: Supplementary file 1 [file ijms-22-05548-s001.zip › ijms-1195487-supplementary.pdf]

Figure S1

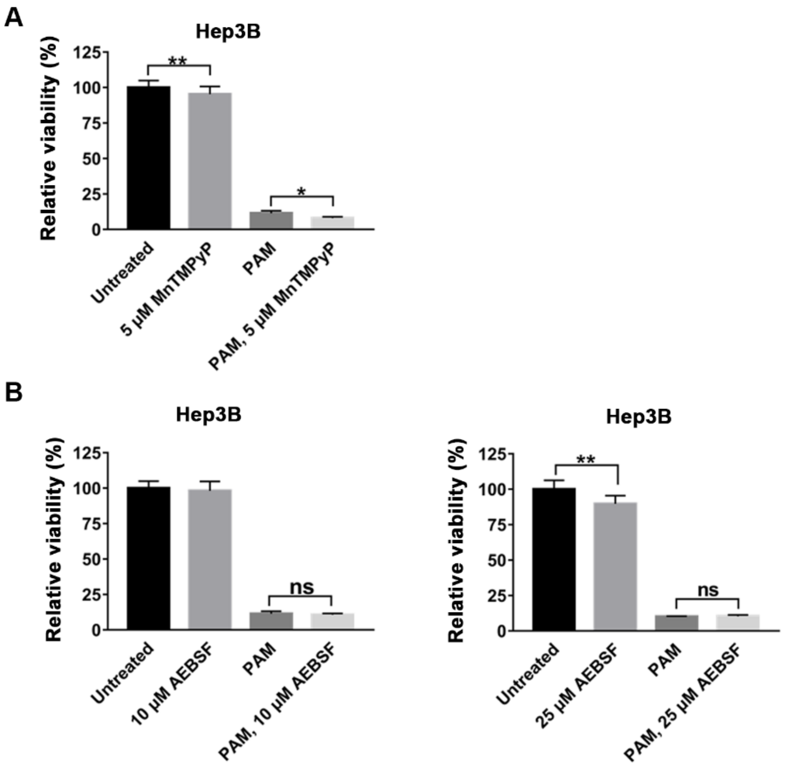

Figure S1. The inhibitors against superoxide anions did not suppress PAM-induced anti-proliferation. (**A**) Hep3B cells were seeded in 35 mm culture dishes and pre-incubated for 18 h. Prior to PAM treatment, the cells were pre-incubated with inhibitors for 1h. Hep3B cells were treated by PAM with the (A) SOD mimetic, 5 μM MnTMPyP, (**B**) NOX1 inhibitor, 10 μM or 25 μM AEBSF. (A, B) Cell viability was analyzed at 72 h after the PAM treatment using MTT assay. The relative viability was calculated as the ratio of the viability of treated cells to that of untreated cells at 72 h. Untreated cells incubated for the same time periods were used as negative controls. The results are presented as the mean ± SD of at least three independent experiments. * $P < 0.05$ indicates significant difference and ns, not significant.

Figure S2

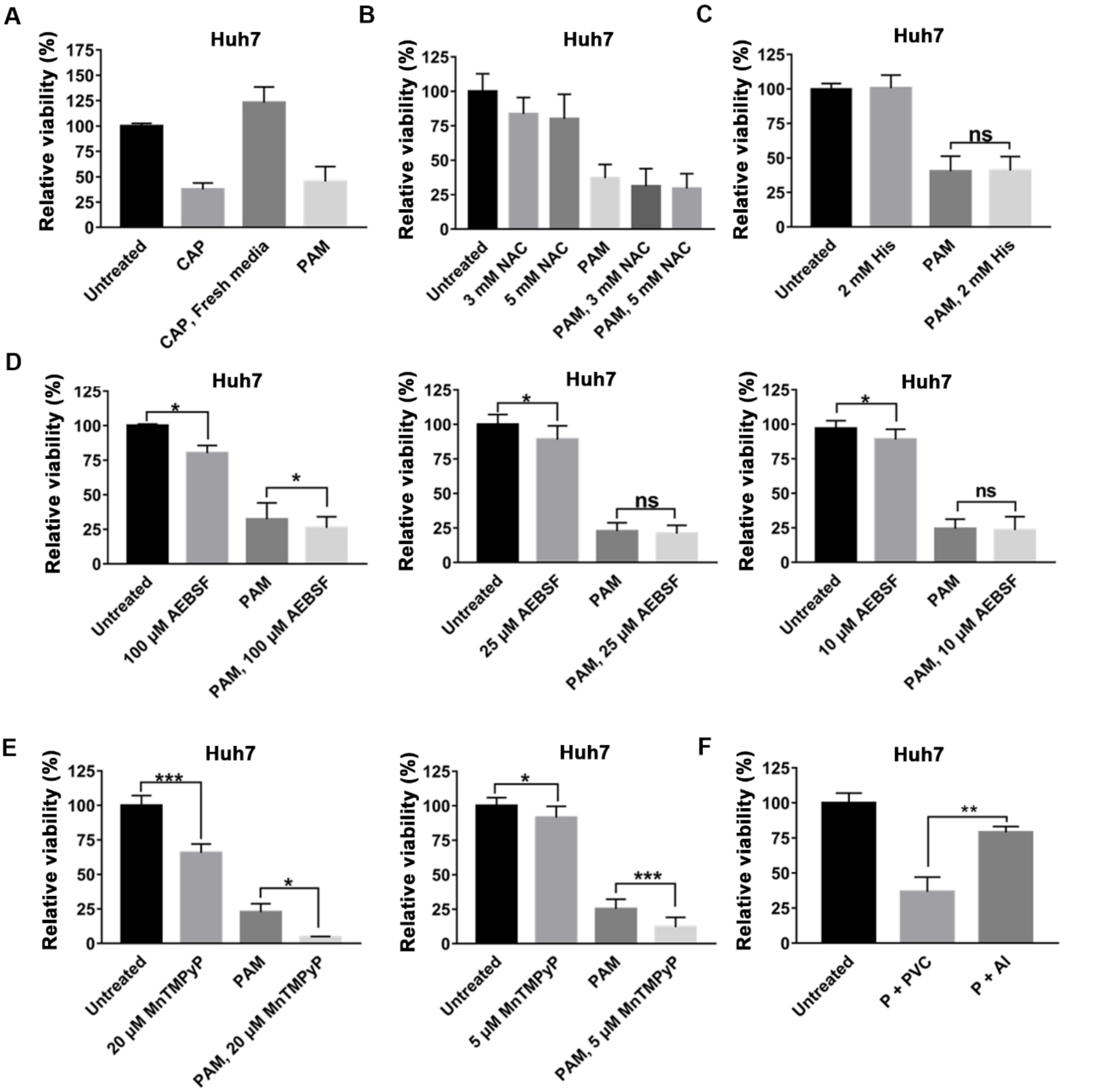

Figure S2. Studies to identify the factors that affect viability in PAM-treated Huh7 cells. (**A**–**F**) Huh7 cells were seeded in 35 mm culture dishes and pre-incubated for 18 h before various treatment. (**A**) The cells were treated with CAP for 2.5 min or PAM or exposed to CAP for 2.5 min followed by an immediate switch to fresh DMEM after the CAP exposure. (**B**) Huh7 cells were treated with 3 mM NAC, 5 mM NAC, PAM, and PAM containing 3 mM or 5 mM NAC. (**C**–**E**) Prior treatment to PAM, the cells were pre-incubated with inhibitors for 1h. Huh7 cells were treated by PAM with the (**C**) $^1O_2$ scavenger, 2 mM histidine, (**D**) NOX1 inhibitor, 100 µM AEBSF, 25 µM AEBSF, and 10 µM AEBSF, (**E**) SOD mimetic, 20 µM MnTMPyP, 5 µM MnTMPyP. (F) The PAM was respectively prepared by placing aluminum mesh, PVC mesh and was treated with Huh7 cells. (**A**–**F**) Cell viability was measured by MTT assay, and the relative viability was calculated as the ratio of the viability of treated cells to the viability of untreated cells at 72 h after the treatment. The results are presented as the mean ± SD of at least three independent experiments. * $p < 0.05$, ** $p < 0.01$, and ***$p < 0.001$ indicate significant difference. ns, not significant.

Figure S3

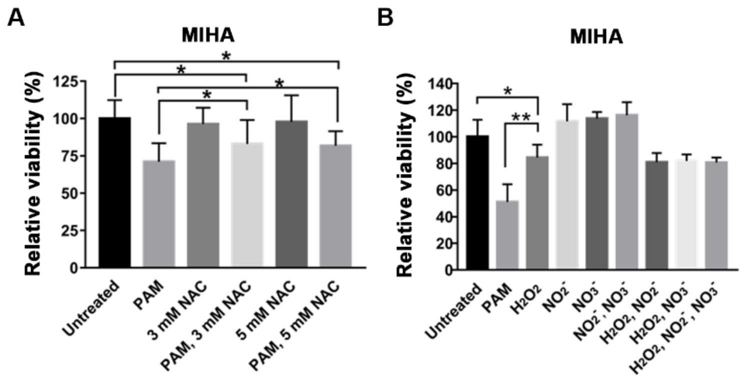

Figure S3. H_2O_2 in PAM decreased the viability of MIHA cells. (**A,B**) MIHA cells were seeded in 35 mm dishes, pre-incubated for 18 h. (**A**) MIHA cells were treated with PAM, 3 mM (or 5 mM), or PAM immediately followed by 3 mM (or 5 mM). (**B**) The effect of NO_2^-, NO_3^- and H_2O_2 individually or in combination on the viability of MIHA cells was compared to the PAM-treated and the untreated control. A 142 μM of H_2O_2, 294 μM of nitrite, and 1.8 mM of nitrate were used. (A, B) The viability was analyzed using MTT assay and the relative viability was calculated as the ratio of the viability of the treated to the untreated at 72 h. The relative viability is presented as the mean ± SD of three independent experiments. * $P < 0.05$ and ** $P < 0.01$ indicate significant difference. ns, not significant.
